# Assessment of awareness and willingness to use pre-exposure prophylaxis for HIV prevention among female sex workers in Rwanda: a cross sectional study

**DOI:** 10.3389/fpubh.2025.1544080

**Published:** 2025-08-20

**Authors:** Albert Nzungize, Athanase Munyaneza

**Affiliations:** ^1^King Faisal Hospital, Kigali, Rwanda; ^2^School of Public Health, University of Rwanda, Kigali, Rwanda; ^3^Research for Development (RD Rwanda), Kigali, Rwanda

**Keywords:** assessment, awareness, willingness to use PrEP, female sex workers, Kigali, Rwanda

## Abstract

**Background:**

Female sex workers (FSWs) in sub-Saharan Africa bear a disproportionate burden of HIV acquisition. While pre-exposure prophylaxis (PrEP) is increasingly accessible in the region, limited data exist on FSWs awareness of and willingness to use PrEP. This study aimed to assess PrEP awareness and willingness to use it, along with associated factors, among FSWs in Kigali, Rwanda.

**Methods:**

This cross sectional study, conducted from April to September 2024, evaluated PrEP awareness and willingness to use it among 333 FSWs over 18 years of age recruited through community based FSW associations. Using a stratified random sampling technique, survey data were analyzed using SPSS, with logistic regression employed to explore associations between participant characteristics and PrEP awareness and willingness. Ethical approval was obtained from the Rwanda National Ethics Committee.

**Results:**

The mean age of participants was 30 years (SD: 7.26), with 47% aged between 25 and 34. Most were single (67%) and unemployed (66%), with nearly half (49.5%) reporting only primary education. A significant proportion (81%) had undergone HIV testing in the past 6 months, and 50% had been involved in sex work for 2 to 5 years. The median number of sexual partners in the past week was 7. Awareness of PrEP was high (81%), however, among those who reported consistent condom use were less likely to be aware of PrEP [adjusted odds ratio (aOR): 0.40, 95% confidence interval (CI): 0.19, 0.83], as were those not screened for sexually transmitted infections (STIs) compared to those who were screened (aOR: 0.43, 95% CI: 0.22, 0.85). Willingness to use PrEP was reported by 80% of participants. Those with a primary education were more willing to use PrEP compared to those with no formal education (aOR: 4.09, 95% CI: 1.62, 10.33). Conversely, participants who were screened for STIs were less likely to report willingness to use PrEP compared to those who were not screened (aOR: 0.28, 95% CI: 0.12, 0.62).

**Conclusion:**

This study demonstrates high awareness and willingness to use PrEP among FSWs in Kigali. However, among those who reported consistent condom use and those unscreened for STIs were less aware of PrEP, and willingness to use it varied based on education and STI screening status. These findings underscore the need for targeted health education and STI screening initiatives to enhance PrEP uptake and strengthen HIV prevention efforts in this vulnerable population.

## Background

HIV remains a critical public health issue, especially among key populations (KPs), such as female sex workers (FSWs). While the global median HIV prevalence among adults aged 15–49 years was 0.8%, it was significantly higher among FSWs, with a median prevalence of 3% in 2023 (1). This highlights the elevated vulnerability of FSWs, with their HIV risk nearly four times that of the general adult population.

In sub-Saharan Africa (SSA), where the HIV prevalence is higher than that in any other global region, data from 2021 revealed that KPs and their sexual partners accounted for 51% of new HIV acquisitions (2). The overall HIV prevalence among adults in Rwanda is 3% (3) and 4.3% in the city of Kigali (3) but is significantly higher among FSWs (51 to 57% in Kigali) (2). The annual HIV incidence is notably elevated for FSWs at 1.36% (4–6).

HIV pre-exposure prophylaxis (PrEP) can substantially decrease new HIV acquisitions, with consistent adherence, reducing the risk of HIV acquisition by nearly 99% (7). Despite a willingness among FSWs in SSA to use PrEP (8–10), multiple barriers impact access and adherence for these populations, including limited awareness of PrEP (8, 11), fear of side effects, social stigma from PrEP use, lack of permission or approval to use from client or partner, fear of perception regarding HIV-positive status (12), and difficulties with daily oral pill use (13) or sex work (14). Additionally, social needs, poverty, and limited access to health care, education and employment are major challenges for FSWs (15, 16). At the health facility level, while trust in certain healthcare providers can positively impact PrEP use for FSWs (17), persistent barriers exist owing to healthcare providers’ limited awareness of PrEP (18).

Although, FSW remains criminalized and highly stigmatized, Rwanda has made significant strides in expanding access to PrEP by 2020, which is a daily tablets containing tenofovir disoproxil fumarate (TDF) with either emtricitabine (FTC) or lamivudine (19), reflecting its commitment to reducing new HIV acquisitions and improving the health of KPs, including FSWs. However, FSWs in the city of Kigali remains at high risk for new HIV acquisitions (6). One study reported that only one-third of the participants consistently used condoms in the past month (5). Despite the potential benefits of PrEP in reducing new HIV acquisitions (20), data on the level of awareness and willingness to use PrEP among FSWs in Rwanda are limited. To address this limitation, we conducted a survey among FSWs in Kigali to assess their awareness of and willingness to use PrEP. The findings from this research provide valuable insights to inform targeted interventions and support the expansion of PrEP services for FSWs in Rwanda.

## Methods

### Study design, setting, and population

This was a cross-sectional study that assessed awareness of and willingness to use PrEP among FSWs in the city of Kigali, Rwanda, between April and September 2024. Rwanda has a population of approximately 13 million (21), with an estimated 8,328–22,806 FSWs, which is predominantly based in Kigali (22). The annual HIV incidence is notably elevated for FSWs at 1.36% (6). The Rwandan guidelines for HIV prevention, treatment and care prioritize KPs for HIV prevention, including FSWs, who are eligible for PrEP if they are HIV negative, are over 18 years of age, and meet specific health criteria (19). PrEP is provided as oral tenofovir disoproxil fumarate-emtricitabine, with clinical evaluations and regular follow-ups to assess adherence and health (19). As of June 2023, the number of FSWs enrollees on PrEP has gradually increased to 10,789 (23).

This study focused on FSWs in major local epidemic areas of Kigali, specifically communities with significantly higher rates of HIV prevalence or incidence than the national average (2, 6). These sites included Nyamirambo in the Nyarugenge district, a densely populated area known for its vibrant community and higher HIV rates among general adults (6); Remera Gisementi in the Gasabo district, characterized by nightlife and entertainment venues that may increase HIV transmission risk; and Kabuga in the Kicukiro district, which contains several neighborhoods with significant numbers of FSWs, contributing to elevated HIV prevalence rates.

### Sampling methods and size

The recent reported size estimate of FSWs in the country, with a median of 13,716, was not stratified by province or by district (22). To determine the sample for this study, using a stratified random sampling technique, we computed a sample size using an independent population. Given that this was a survey, our confidence interval was set at 95%, corresponding to a Z score of 1.96 and a margin of error of 5%. With this, given Z = 1.960, *p* = 0.5, and M = 0.05. The sample size formula was S = Z^2^ × P × [(1-P)/M ^2^]. S = (1.960)^2^ × 0.5 × [(1–0.5)/0.05^2^] = (3.8416 × 0.25)/0.0025.

S = 384.16 ~ 384 were equally distributed across the three study areas.

### Data collection

We collaborated with FSWs associations in the neighborhoods of each study site, engaging FSWs who had previously worked with nongovernmental organizations (NGOs) experienced in health promotion. Each site had a designated head of association and an FSWs known for collaborating with other NGOs. Study participant recruitment took place three times a week—on Friday, Saturday, and Sunday evenings—during times recommended by the heads of FSWs associations, when most peers were present at the site. Only FSWs who presented at the study site for reasons unrelated to the study and were willing to speak with the selected heads of FSWs associations were recruited to participate if potentially eligible. Prior to data collection, eligible participants had to self- identified as FSWs, aged 18 years and above and willing to provide written consent. Also*, Questions were evaluated by a national expert in the field prior to administration, followed by a pilot test with ten participants to assess clarity and understanding.* In person survey data collection was conducted in Kinyarwanda by an experienced researcher and study assistant, both of whom are public health specialists. The participants who completed the survey received a cash incentive of 5,000 Rwandan francs (approximately $3.50) to compensate for opportunity costs and transportation expenses. The survey lasted between 1 and 2 h per participant. Additionally, the heads of FSWs associations helped create a conducive environment for efficient and convenient data collection. To ensure quality assurance, weekly meetings were held with data collectors, the study coordinator, and the co-investigator to address data collection challenges, discuss solutions, and review the collected paper-based data before the survey forms were handed over to the data entry team.

### Study variables and measurements

The primary objective of the study was to assess the following: Awareness of PrEP: Defined as participants responding “yes” to the question, “Before today, have you ever heard of HIV-uninfected people taking ARV every day to reduce the risk of getting HIV?” Willingness to use PrEP: Defined as participants indicating their willingness to “take ARV every day to lower the chances of contracting HIV.” Additional variables included the following: sociodemographic information, data on participants’ age, marital status, employment status categorized as “no paid job, housewife, market vendor and others (none specified, student and security),” and highest level of education. Sexual history and HIV risk: Information gathered included the time since the last HIV test, the time since sex work started, the number of sexual partners in the 7 days before the survey, and the frequency of condom use. STI screening and family planning methods: Collected data on whether participants had been screened for STIs in the past 12 months and the use of family planning methods. Table 1 shows the structure of the survey questions and the categorization of the variables.

**Table 1:** Survey questions and variable categorizations.

Number	Survey questions	Categorization
1	What is your age?	1. 18–24 years
		2. 25–34 years
		3. 35–44 years
		4. 45–54 years
2	What is your marital status?	1. Single
		2. Married/living with
		3. Separated
3	What is your main activities?	1. No paid job
		2. Housewife
		3. Market vendor
		4. Others (other, student and security)
4	What is your highest level of education?	1. None
		2. Primary
		3. Secondary/vocational
5	When is your last HIV test?	1. < 6 months
		2. > = 6 months
6	Since when did you start being sex workers?	1. <= 1 years
		2. 2–5 years
		3. 6–10 years
		4. > 10 years
7	How many partner have you had vaginal sex in the past 7 days and past 12 months … 7 days	1. < 5 partners
		2. 5–10 partners
		3. 11–20 partners
		4. > 20 partners
8	How often are condoms used during vaginal sex for the last 7 days?	1. Never (none, did not have sex)
		2. Always
		3. Sometimes
9	Do you use any method to delay or avoid pregnancy?	1. None
		2. Hormonal (pills. Implant, injectable)
		3. Others (IUD, ligature)
10	Have you ever screened for STIs in the past 12 months?	1. Yes (screened)
		2. Not screened
11.	Before today, how much would you say you knew about PrEP?	0. Not aware of PrEP
		1. Aware of PrEP (all yes)
12	Based on this introduction, do you think you could benefit from PrEP?	1. Yes
		2. No
13	PrEP is now available free of charge at health centers. Would you be willing to start taking PrEP in the next month to protect yourself against HIV?	0. Not willing to use (no, and I do not know)
		1. Willing to use (all yes)

### Data analysis

The data were analyzed via IBM SPSS Statistics (version 21). Frequencies and proportions were calculated for categorical variables with complete data, whereas means or medians were used for continuous variables. For the outcome variable PrEP awareness, responses were dichotomized as ‘Yes’ (‘Yes, I have heard of PrEP,’ ‘Yes, I know what PrEP is’) or ‘No’ (‘No, I have never heard of PrEP,’ ‘No, I do not know what PrEP is’). For willingness to use PrEP, responses were dichotomized as ‘Yes’ or ‘No’ (‘No, I do not know’). The mean age, median duration as an FSWs, and number of sexual partners in the previous 7 days were also calculated.

Bivariate logistic regression, accounting for the number of participants to the awareness and willingness questions, was used to examine associations between individual characteristics and these outcomes. The results are reported as unadjusted odds ratios (ORs) with 95% confidence intervals (CIs). Using stepwise selection, the multivariable logistic regression model included individual characteristics that were significantly associated with the outcomes (*p* < 0.05) in the bivariate analysis. Associations are reported as adjusted odds ratios (aORs) with 95% CIs at a significance level of 0.05 for PrEP awareness and willingness to use.

### Ethical considerations

This study was approved by the Rwanda National Ethics Committee (RNEC 76/2024), which also approved the informed consent forms. Written informed consent was obtained from all participants prior to their inclusion in the study. Participants were provided with detailed information about the study objectives, procedures, potential risks, and benefits. They were assured of their right to withdraw at any time without any consequences. Confidentiality of all personal data was guaranteed throughout the study, only study staff had access to the data.

## Results

**Figure 1:**
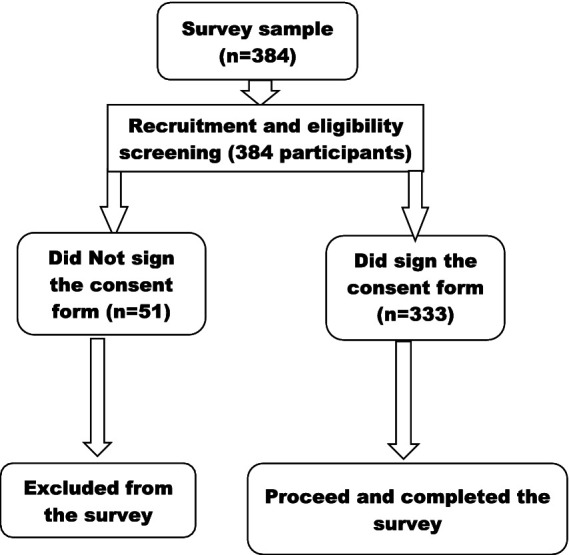
Participant flow in the study survey.

Among the 333 participants in this study (refer to data flow Figure 1), the majority of participants were single (67%), unemployed in the formal workforce (66%), and held a primary school-level education (49.5%), with a mean age of 30 years (Table 2). Approximately 81% (258) of the sample reported having had an HIV test within 6 months prior to the survey, 59% reported being screened for an STI in the last 12 months, and 50% (165) had been engaged in sex work for 2–5 years. The median number of sexual partners in the last 7 days was 7, with approximately 40% (132) reporting fewer than 5 partners during that period, approximately 49% (162) indicating the use of condoms sometime in the past week, and 72.5% indicating that they were using hormonal contraception at the time of the survey.

**Table 2:** Survey participant characteristics (*n* = 333).

FSWs characteristics	Frequency (N), median	Percentage
Age categories		
Mean age	30 years	
18–24 years	77	23.1
25–34 years	156	46.8
35–44 years	91	27.3
45–54 years	9	2.7
Current marital status		
Single	222	66.7
Married/living with male partner	23	6.9
Separated	88	26.4
Employment status		
No paid job	218	65.7
Housewife	25	7.5
Market vendor	58	17.5
Others (other, student and security)	31	9.3
Education level		
None	96	29.2
Primary	163	49.5
Secondary/vocational	70	21.3
Time of Last HIV Test		
< = 6 months	258	81.4
> = 6 months	59	18.6
Duration since initiation of sex work (years)		
Median time	5 years	
<= 1 years	18	5.5
2–5 years	165	50.0
6–10 years	96	29.1
> 10 years	51	15.5
Number sex partners in the last 7 days		
Median number	7 partners	
< 5 partners	132	40.0
5–10 partners	113	34.2
> = 11 partners	85	25.8
Condom use in the prior 7 days among those who had sex		
Sometimes	162	48.9
Always	141	42.6
Never (none, did not have sex)	28	8.5
Screen for STIs in the past 12 months		
Yes	190	58.3
No	136	41.7
Utilization of family planning methods		
None	84	25.4
Hormonal Contraception methods	240	72.5
Others (IUD, ligature)	7	2.1
Perceived PrEP benefit		
Yes	252	95.1
No	13	4.9
PrEP awareness		
Not aware of PrEP	51	18.8
Aware of PrEP	220	81.2
Willingness to use PrEP		
No	54	20.3
Yes	212	79.7

### Awareness of and willingness to use PrEP

There was a high level of reported awareness of PrEP (81%) and the benefit of using PrEP to reduce the risk of HIV (95%) among the sample. In the bivariate analysis, PrEP awareness was significantly associated with condom use in the prior 7 days, STI screening in the past 12 months, and perceived PrEP benefit (Table 3).

**Table 3:** Bivariate analysis of survey characteristics and awareness of and willingness to use PrEP among FSWs.

FSWS characteristics	Bivariate analysis			
	PrEP awareness		Willingness to use PrEP	
	Aware of PrEP (*n*)	OR (95% CI)	Willing to use (*n*)	OR (95% CI)
Age categories				
18–24 years	53	Ref	56	Ref
25–34 years	104	1.14 (0.54, 2.39)	102	0.83 (0.38, 1.82)
35–44 years	59	1.55 (0.63, 3.80)	50	0.61 (0.26, 1.44)
45–54 years	4	0.35 (0.07, 1.76)	4	0.26 (0.05, 1.33)
Current marital status				
Single	158	Ref	154	Ref
Married/living with male partner	14	0.70 (0.22, 2.29)	11	0.40 (0.14, 1.17)
Separated	48	0.65 (0.32, 1.29)	47	0.74 (0.36, 1.49)
Employment status				
No paid job	136	Ref	131	Ref
Housewife	19	0.74 (0.27, 2.01)	16	0.48 (0.19, 1.24)
Market vendor	47	1.84 (0.72, 4.68)	46	1.60 (0.68, 3.88)
Others (other, student and security)	17	0.57 (0.21, 1.49)	18	0.62 (0.24, 1.63)
Education level				
None	66	Ref	56	Ref
Primary	105	1.43 (0.70, 2.90)	114	**5.08 (2.28, 11.32)**
Secondary/vocational	46	1.04 (0.46, 2.37)	40	1.05 (0.50, 2.19)
Time of Last HIV Test				
<= 6 months	173	Ref	167	Ref
> = 6 months	38	1.75 (0.65, 4.74)	33	0.83 (0.37, 1.81)
Duration since initiation of sex work (years)				
<= 1 years	9	Ref	9	Ref
2–5 years	114	2.87 (0.88, 9.41)	113	2.39 (0.67, 8.48)
6–10 years	61	1.99 (0.59, 6.74)	59	1.38 (0.38, 4.99)
> 10 years	36	2.85 (0.73, 11.13)	31	1.37 (0.34, 5.45)
Number sex partners in the last 7 days among those who had sex				
< 5 partners	70	Ref	62	Ref
5–10 partners	85	1.99 (0.95, 4.16)	81	**038 (0.17, 0.83)**
> = 11 partners	48	1.50 (0.71, 3.16)	69	0.80 (0.35, 1.85)
Condom use in the prior 7 days				
Sometimes	110	Ref	104	Ref
Always	88	**0.38 (0.19, 0.76)**	91	0.63 (0.33, 1.23)
Never (none, did not have sex)	22	0.60 (0.19, 1.82)	17	**0.34 (0.13, 0.88)**
Screen for STIs in the past 12 months				
Yes	137	Ref	137	Ref
No	83	**046 (0.29, 0.85)**	75	**0.27 (0.14, 0.51)**
Utilization of Family Planning Methods				
None	53	Ref	44	Ref
Hormonal Contraception methods	164	1.36 (0.69, 2.70)	165	**2.34 (1.22, 4.49)**
Others (IUD, ligature)	3	0.42 (0.6, 2.77)	3	0.68 (0.10, 4.40)
Perceived PrEP benefit				
Yes	206	Ref	201	Ref
No	7	**0.23 (0.07, 0.74)**	4	**0.10 (0.03, 0.38)**

Ref: reference category, bold variables: variables with statistical significance.

According to the multivariable logistic regression analysis (Table 4), FSWs who reported always using condoms during sex in the prior 7 days were less likely to be aware of PrEP (aOR: 0.40, 95% CI: 0.19–0.83) than those who sometimes used condoms. Similarly, FSWs who had not been screened for STIs in the past 12 months were less likely to be aware of PrEP (aOR: 0.43, 95% CI: 0.22–0.85) than those who had been screened. Additionally, FSWs who did not perceive a benefit from PrEP were less likely to be aware of it (aOR: 0.20, 95% CI: 0.05–0.67) than those who recognized its benefit.

**Table 4:** Multivariable analysis of survey participants’ characteristics and awareness of, and willingness to use, PrEP.

FSWs characteristics	Multivariable analysis			
	PrEP awareness		Willingness to use PrEP	
	Aware of PrEP (*n*)	aOR (95% CI)	Willing to use (*n*)	aOR (95% CI)
Education level				
None			56	Ref
Primary			114	**4.09 (1.62, 10.33)**
Secondary/vocational			40	0.76 (0.30, 1.92)
Number sex partners in the last 7 days				
< 5 partners			62	Ref
5–10 partners			81	1.46 (0.62,3.45)
> = 11 partners			69	1.27 (0.46, 3.49)
Condom use in the prior 7 days among those who had sex				
Sometimes	110	Ref	104	Ref
Always	88	**0.40 (0.19, 0.83)**	91	0.80 (0.35, 1.82)
Never (none, did not have sex)	22	0.82 (0.25, 2.72)	17	0.95 (0.26, 3.44)
Screen for STIs in the past 12 months				
Yes	137	Ref	137	Ref
No	83	**0.43 (0.22, 0.85)**	75	**0.28 (0.12, 0.62)**
Utilization of family planning methods				
None			44	Ref
Hormonal Contraception methods			165	1.53 (0.70, 3.32)
Others (IUD, ligature)			3	1.38 (0.13, 14.56)
Perceived PrEP benefit				
Yes	206	Ref	201	Ref
No	7	**0.20 (0.05, 0.67)**	4	**0.06 (0.16, 0.29)**

Ref: reference category, bold variables: variables with statistical significance.

Among FSWs participants, 80% (212) reported being willing to use PrEP in the next month to protect themselves against HIV. In the bivariate analysis, willingness to use PrEP was significantly associated with education level, condom use in the previous 7 days, STI screening in the previous 12 months, the number of sexual partners in the previous 7 days, the use of family planning methods, and perceived PrEP benefits (Table 3). According to the multivariable logistic regression model, FSWs with a primary education level were more likely to be willing to use PrEP (aOR: 4.09, 95% CI: 1.62–10.33) than were those with no formal education. Additionally, FSWs who had not been screened for STIs in the past 12 months were less likely to be willing to use PrEP (aOR: 0.28, 95% CI: 0.12–0.62) than those who had been screened. Furthermore, FSWs who did not perceive a benefit from PrEP were less likely to be willing to use it (aOR: 0.06, 95% CI: 0.16–0.29) than those who recognized its benefit.

## Discussion

In this study, we assessed awareness of and willingness to use PrEP among FSWs in three major HIV epidemic areas of Kigali. We found high levels of awareness and willingness to use within this population.

High awareness of PrEP among FSWs has been reported in SSA. For example, a cohort of 700 HIV-negative FSWs in Dar es Salaam reported a PrEP awareness rate of 67% at enrollment of the cohort, which increased to 97% after 12 months (8). Similarly, in Nigeria, a cross-sectional study of 344 FSWs receiving health promotion and prevention services at the One Stop Shop (OSS) reported a PrEP awareness rate of 76% (24), whereas an online survey reported a PrEP awareness rate of 95% (15). While evidence on PrEP awareness among FSWs in Rwanda remains limited, one of our studies reported that 62% of FSWs were of PrEP (25), whereas a study in Nigeria reported lower awareness; for example, a cross-sectional study in Anambra State reported an awareness rate of only 31% (26). These differences may be attributed to our convenience sampling of FSWs from major HIV epidemic areas in Kigali. This study was conducted 2 years later, reporting 62% PrEP awareness among FSWs (25). While the previous study used routine clinical data from a primary health facility, our study collected data from major HIV epidemic areas where FSWs networks are likely stronger. In contrast, the Anambra study involved brothel FSWs recruited through the snowball technique. Notably, our prior study assessing PrEP awareness among men who have sex with men (MSM) in Rwanda—a KP also eligible for PrEP—reported similarly high awareness levels to our findings (27), suggesting that access to PrEP information may be consistent across KPs in the country.

Awareness of PrEP was lower among FSWs who reported always using condoms than among those who used them less frequently. It was also lower among those who had not been screened for STIs in the past 12 months compared to those who had, and among those who did not perceive the benefits of PrEP compared with those who did. While these findings indicate high awareness among FSWs, they also highlight the need for targeted educational interventions to increase PrEP awareness within specific subgroups. Our findings indicate that FSWs who reported always using condoms had lower awareness of PrEP than did those who did not. This may suggest that condom users believe that their risk of HIV transmission is adequately reduced by condom use alone, resulting in low PrEP usage as an additional preventive measure. Similarly, a research conducted in Kenya revealed that PrEP users often questioned the necessity of combining PrEP with condoms, believing that PrEP alone sufficiently reduced their HIV risk (28). This perspective sometimes led to frustration with healthcare providers who recommended continued condom use alongside PrEP. Public health programs should address the misconception that PrEP is only for those who use condoms inconsistently. Messaging should emphasize that PrEP provides an additional layer of protection even for those who consistently use condoms.

Furthermore, our findings revealed that FSWs who did not perceive any benefit from PrEP were significantly less likely to be aware of it. This highlights the crucial role of perceived benefits in shaping health behavior. Those who do not view PrEP as useful may not seek information or education about it, leading to lower levels of awareness. This lack of perceived benefit could stem from a misunderstanding of how PrEP works, a belief that other preventive measures (such as condoms) are sufficient, or misinformation about the effectiveness or side effects of PrEP. Without understanding the value that PrEP can provide in preventing HIV, FSWs may not engage with or prioritize information about it, resulting in lower awareness. Similarly among another group of KP in developed country reliance on condoms led many to view PrEP as unnecessary, reducing their interest in learning about or using it (29).

Our survey findings indicated a high willingness to use PrEP among FSWs, particularly among those with a primary education level, compared with those with no formal education. Furthermore, FSWs who had not undergone STI screening in the past 12 months were significantly less likely to be willing to use PrEP, as were those who did not perceive a benefit from it. These results emphasize the critical role of education, STI screening, and the perception of the benefits of PrEP in influencing the willingness to adopt PrEP among this population. Although no studies on PrEP willingness among FSWs in Rwanda have been conducted, previous research among MSM in the country reported a high willingness to use PrEP, exceeding 80% (9, 27).

Additionally, our findings showed that FSWs who had not been screened for STIs in the past 12 months were less likely to be willing to use PrEP. Other studies have reported that STI screening is associated with increased odds of willingness to use PrEP (28, 29), reinforcing the idea that regular health visits increase exposure to valuable health information. These findings suggest that regular STI screenings may provide additional health information, including information on HIV prevention methods such as PrEP. Those who were not screened may have avoided health facilities, lacked interest in PrEP information, or relied on other HIV prevention methods, such as condoms.

The high willingness to use PrEP reported in our findings suggests promising potential for PrEP uptake in this population, which could help reduce HIV acquisition in high-risk areas. A scoping review of PrEP adherence reported that the success of PrEP programs depends on willingness to use and prior awareness (12). Therefore, the high reported willingness to use PrEP among FSWs concentrated in major HIV epidemic areas in Kigali suggests a greater likelihood of PrEP uptake in this population.

Similar findings have been reported across SSA, where high levels of willingness to use PrEP were observed. For example, in a cohort study conducted in Tanzania, 98% of the participants were willing to use PrEP at enrollment, whereas 96% remained willing after 12 months (8). In another study in Anambra State, Nigeria and Uganda, 91% of participants expressed a strong willingness to use PrEP (26, 30). Furthermore, a study in Ghana reported a willingness to use PrEP rate of 80% among FSWs (31). These results demonstrate a consistent trend of high acceptance and willingness to use PrEP in different regions of SSA.

Our study revealed that FSWs with a primary education level were more likely to express willingness to use PrEP than were those with no education. This highlights the potential influence of education on PrEP uptake and the need for targeted interventions in less educated populations. A study among adolescent girls in Rwanda has indicated an association between education level and general knowledge of HIV acquisition (32). Although this study did not focus specifically on FSWs, its findings suggest that higher education levels are associated with increased odds of having comprehensive HIV knowledge, including preventive measures. These results align with our findings, where FSWs with at least one primary education level were more willing to use PrEP. Similar studies, such as one in Nigeria (26), showed that FSWs seeking more HIV knowledge had higher odds of PrEP willingness, and research in Uganda also reported that education increases the willingness to use PrEP (33).

An emerging body of literature demonstrates that increasing PrEP awareness among FSWs is a key predictor of higher uptake and sustained long-term engagement with the intervention (8, 33).

Our findings showed that FSWs who did not perceive any benefit from PrEP were less likely to be willing to use it. This underscores the need for targeted education and awareness campaigns that emphasize the effectiveness of PrEP in preventing HIV, particularly among high-risk populations. This suggests the need to integrate comprehensive PrEP education into existing sexual health services for FSWs while ensuring that healthcare providers are trained to communicate the benefits of PrEP effectively. Additionally, addressing misconceptions and barriers to understanding the value of PrEP could significantly increase its uptake among FSWs. Similarly, a qualitative study in Morocco found that doubts about PrEP effectiveness and concerns about side effects hindered its uptake among FSWs, highlighting the need for targeted education (34).

## Limitations

The findings from this survey have several limitations. As a cross-sectional study, it could not report changes in awareness or willingness to use PrEP over time, which is essential for predicting significant uptake. Future studies could follow up on this assessment.

Additionally, the survey included FSWs who self-reported not currently using PrEP; however, it did not ask whether they had used it before, nor could it verify the accuracy of these self-reports.

Furthermore, the survey was conducted during a period when the PrEP rollout project was being implemented across most primary health facilities in Rwanda. This may have resulted in more information being available to eligible individuals, including FSWs, contributing to the observed high awareness of PrEP.

Although the target sample included 384 participants, only 333 participants were analyzed because of incomplete responses, missing data, and unmet inclusion criteria. Nonetheless, the analyzed sample remains sufficient for meaningful analysis.

Additionally, a limitation of the study is that data analysis was conducted by a single author, which may introduce bias. Future analyses could involve multiple authors to ensure a more balanced interpretation.

Finally, the survey focused on major HIV epidemic areas in Kigali, which limits the generalizability of the findings to other regions in the country.

Despite these limitations, the survey has notable strengths, this includes providing data on PrEP awareness and willingness to use it among FSWs, where such data are currently limited, particularly in Rwanda.

## Conclusion

This survey assessed awareness of and willingness to use PrEP among FSWs in Kigali, Rwanda. The findings revealed high levels of both awareness and willingness, indicating strong potential for PrEP uptake within this KP. These insights are valuable for informing targeted interventions aimed at expanding PrEP services in Rwanda. However, certain subgroups—such as FSWs who consistently use condoms, those with limited STI screening history, and those who perceive low benefit from PrEP—may require tailored strategies to enhance engagement. To support broader acceptance and adoption of PrEP, interventions should emphasize education, regular health screenings, and outreach campaigns highlighting the benefits of PrEP alongside other HIV prevention methods. These efforts will help ensure that all FSWs, regardless of individual risk perception or current prevention practices, are empowered with comprehensive HIV prevention options.

## Data Availability

The raw data supporting the conclusions of this article will be made available by the authors, without undue reservation.
